# Recurrent polyploidy and descending dysploidy as plant genome shapers: Insights from Sporobolus (Chloridoideae, Poaceae) genomes

**DOI:** 10.1371/journal.pone.0343073

**Published:** 2026-02-23

**Authors:** Morgane Milin, Oscar Lima, Lin-Feng Li, Bo Li, Marc Beringer, Christian Parisod, Malika Ainouche, Armel Salmon

**Affiliations:** 1 UMR CNRS 6553 ECOBIO, University of Rennes, Campus de Beaulieu, Rennes Cedex, France; 2 State Key Laboratory of Biocontrol, Guangdong Provincial Key Laboratory of Plant Resources, School of Life Sciences, Sun Yat-sen University, Guangzhou, China; 3 State Key Laboratory of Wetland Conservation and Restoration, National Observations and Research Station for Wetland Ecosystems of the Yangtze Estuary, Ministry of Education Key Laboratory for Biodiversity Science and Ecological Engineering, Institute of Eco-Chongming, School of Life Sciences, Fudan University, Shanghai, China; 4 State Key Laboratory for Vegetation Structure, Functions and Construction, Ministry of Education Key Laboratory for Transboundary Ecosecurity of Southwest China, Yunnan Key Laboratory of Plant Reproductive Adaptation and Evolutionary Ecology, Institute of Biodiversity, School of Ecology and Environmental Science and the Southwest United Graduate School, Yunnan University, Kunming, China; 5 Department of Biology, University of Fribourg, Fribourg, Switzerland; University of Guelph, CANADA

## Abstract

Polyploidization or whole genome duplication (WGD) is a source of genetic and phenotypic novelties and is a widespread mechanism of speciation across plant lineages. It is often followed by complex genome dynamics, including diploidization. Recurrent polyploidization leads to overlapping genomic processes complicating efforts to reconstruct genome histories in extant species. Here, we focused on the complex and understudied Chloridoideae subfamily of grasses, where polyploidy is recurrent and base chromosome number variation particularly common. We explored the evolutionary history of *Sporobolus* genomes through comparative genomics analyses, including species from sections *Sporobolus* and *Spartina* and selected representatives of different grass lineages. We used the WGD_Tracker pipeline to identify homologous genes and estimate their divergence, as well as to detect syntenic regions, and reconstruct karyotypes. We found that sections *Sporobolus* and *Spartina* diverged 13.2–26.0 million years ago (Mya), based on molecular clock analyses, and showed that the two WGD events detected in section *Spartina* (*S. maritimus* and *S. alterniflorus*) occurred independently of another WGD in section *Sporobolus* (*S. stapfianus* and *S. pyramidalis*). We also identified five nested chromosome insertions (NCI), a major descending disploidy mechanism that resulted in a new base chromosome number (n = 15) in section *Spartina* subsection *alterniflori*. The ancestral grass chromosome 12 appears particularly prone to structural modifications – such as insertions and rearrangements – throughout Chloridoideae evolution. Both ancestral chromosomes 11 and 12 were involved in a recent rearrangement that contributed to chromosome number divergence during speciation between *S. alterniflorus* (2n = 62) and *S. maritimus* (2n = 60), estimated at 3.7–7.7 Mya. Comparative analyses of Chloridoideae genomes provide new insights into genome duplication histories and post-polyploidization genome restructuring through descending dysploidy and revealed that NCIs are a prevalent diploidization mechanism, offering new perspectives to explore the genomic innovations underpinning the success of allo-polyploids.

## Introduction

Plant genome dynamics is shaped by recurring cycles of polyploidization and diploidization processes, as widely documented across angiosperms [1,2]. Polyploidization or whole genome duplication (WGD) results in the presence of more than two chromosome sets in the nucleus. It has long been recognized as a major evolutionary process in plants, leading to the formation of new species [3–5].

Diploidization is the process that returns a polyploid genome to a diploid-like state by restoring bivalent pairing and disomic inheritance [6]. Diploidization is driven by a variety of cytological and genetic mechanisms [6], such as gene fractionation (i.e., duplicated gene loss) [7–9], descending dysploidy (i.e., chromosome number reduction) [10], or genome downsizing (i.e., genome size reduction) [11–13]. Superimposed WGD events dating back to ancestors of various plant lineages were documented, and extant diploid species are now considered to be diploidized paleo-polyploids [14,15]. Consequently, much attention has been paid to the formation pathways of polyploids, and to the immediate genetic, phenotypic, and ecological consequences of polyploidy [16–21]. However, much remains to be understood regarding the frequency and nature of these phenomena across the various plant lineages, especially in non-model taxa.

The Poaceae family is an excellent model for investigating polyploid genome evolution. It is one of the most polyploid-rich plant families (*~*80% of species) [22,23], shaped by an ancient shared WGD event (*rho*, ≈ 90–115 million years ago) [24,25], as well as several more recent lineage-specific WGDs [8,26] that have contributed to its adaptive diversification and evolutionary success [27].

Subfamilies of Poaceae containing key crop species are particularly well studied in terms of genome evolution. For example, the rice genome (*Oryza sativa*, Oryzoideae, 2n = 2x = 24), which has not experienced any WGD since the *rho* event, serves as a reference for grass comparative genomics due to its highly conserved ancestral genome structure [24, 28]. In the Panicoideae subfamily, chromosome fusions led to a reduction in basic chromosome number from 12 to 10, as observed in the genomes of *Sorghum bicolor* (2n = 2x = 20) [29] and *Zea mays*. The latter experienced a WGD 5–12 million years ago (Mya) [30,31], followed by chromosome fusions and transposable elements proliferation, shaping a diploidized meso-tetraploid (2n = 2x = 20) modern genome [32]. In *Saccharum spontaneum* (2n = 40–128), chromosome rearrangements led to new basic chromosome numbers of x = 8 and x = 9, followed by additional polyploidization events [33].

In the Pooideae subfamily, genome duplication and chromosome rearrangements have been well-documented, most particularly in the Triticeae tribe [34]. Nested chromosome insertions (NCI) were reported as the main mechanism involved in descending dysploidy in this tribe, and involved in a chromosome number reduction from 12 to 7 [35]. Various WGDs have been identified in different lineages, such as in the allo-hexaploid wheat (*Triticum aestivum*, AABBDD, 2n = 6x = 42) that resulted from two successive allo-polyploidization events <0.8 and <0.4 Mya, respectively [36].

Despite extensive work in Oryzoideae, Panicoideae, and Pooideae, Chloridoideae remain underexplored. This subfamily includes 1,603 described species distributed in 121 genera and five tribes (Centropodieae P.M. Peterson, Triraphideae P.M. Peterson, Eragrostideae Stapf., Zoysieae Benth. and Cynodonteae Dumort.; [37]), and exhibits highly variable basic chromosome numbers (x = 6, 7, 8, 9, 10) [38] and ploidy levels ranging from 2x to 20x [39]. Chromosome scale assemblies were recently reported in Eragrostideae (*Eragrostis curvula* [40]; *E. tef* [41]), Cynodonteae (*Cynodon dactylon* [42]; *C. transvaalensis* [43]; *Eleusine coracana* [44]; *Oropetium thomaeum* [45]) and Zoysieae (*Zoysia japonica* [46]; *Sporobolus alterniflorus* [47]; *S. maritimus* [48]) tribes. Two chromosomal fusion events were reported as ancestral in Chloridoideae, resulting in a chromosome number reduction from 12 to 10 [42,44,46].

In this study, we focus on the large and taxonomically complex *Sporobolus* genus for which chromosome number and ploidy level information are still lacking for many species. Four genomes are sequenced to date: *S. stapfianus* and *S. pyramidalis* in section *Sporobolus* [49], *S. alterniflorus* [47] and *S. maritimus* [48] in section *Spartina*. Phylogenetic analyses revealed the paraphyletic nature of the former *Sporobolus* genus and proposed the inclusion of the former *Calamovilfa* and *Spartina* genera (where they are now considered as sections) [50].

The sect. *Spartina* represents a monophyletic clade that diversified within the last 10 million years [51]. This section represents a model in evolutionary ecology with several foundation species in saltmarsh ecosystems [52]. *S. alterniflorus* and *S. maritimus* are considered as “ecosystem engineers” on salt marshes [53,54]. Section *Spartina* is a model system in plant speciation research, with a textbook example of neo-polyploidy, following the hybridization between *Sporobolus alterniflorus* (2n = 62) and *Sporobolus maritimus* (2n = 60) during the 19^th^ century which resulted in the formation of the highly invasive allo-polyploid, *Sporobolus anglicus* (2n = 124) [55]. As the basic chromosome number in this group was traditionally considered to be x = 10 [56], *S. maritimus* and *S. alterniflorus* were considered hexaploid, and their descendant *S. anglicus* as allo-dodecaploid [57]. The allo-polyploid nature of the parental species was hypothesised according to the propensity of interspecific hybridization in the sect. *Spartina* and to the detected presence of divergent duplicated genes in both *S. maritimus* and *S. alterniflorous* [58,59]. However, recent whole genome analyses of the parental species [48] revealed that both genomes have been shaped by two successive WGDs: the first event dating back to 9.6–24.4 Mya (WGD1) led to a tetraploid genome with 2n = 4x = 40 which was followed by a basic chromosome number reduction from n = 20 and x = 10 to n = x = 15. The second event dating back to 2.1–6.2 Mya (WGD2) led to n = 30, with two sets of 15 homeologous subgenomes. *S. maritimus* and *S. alterniflorus* then appear to be tetraploids that diverged following a chromosomal rearrangement in the *S. alterniflorus* ancestor [48]. According to the estimated divergence times of these events, WGD1 occurred before the emergence of the section *Spartina* species.

In this paper, we explore the evolutionary history of *Sporobolus* genomes, focusing on the evolution of chromosomal structure. The following questions are addressed: (i) What is the structure of available *Sporobolus* genomes compared to the ancestral Poaceae genome? (ii) Are WGD events reported to date in *Sporobolus* shared with other Chloridoideae genomes? (iii) What are the mechanisms responsible for basic chromosome number changes?

**Fig 1:**
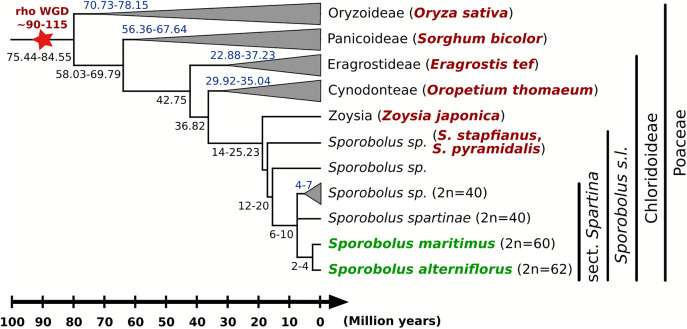
Phylogenetic relationships among grass species (highlighted in red), compared to *Sporobolus maritimus* and *S. alterniflorus* (in green), as analyzed in this study. Divergence times are derived from [51,60], while estimates of the *rho* whole genome duplication (WGD) are from [24,25].

To address these questions, we conducted comparative genome analyses involving *Sporobolus* species from sections *Sporobolus* and *Spartina*, along with representative species from key Poaceae lineages (e.g., Oryzoideae, Pooideae, Panicoideae, Chloridoideae; Fig 1).

## Materials and methods

### Selected set of Poaceae species for comparative genomics

We compared the recently sequenced genome of *Sporobolus maritimus* (2n = 4x = 60; Chloridoideae, Zoysieae, *Sporobolus* sect. *Spartina*) [48] and *Sporobolus alterniflorus* (2n = 4x = 62) [47] with a curated panel of grass genomes selected on the basis of phylogenetic proximity and genome assembly quality (Fig 1; S1 Table). The panel included: the two closest phylogenetic relatives, *Sporobolus stapfianus* (4x) and *Sporobolus pyramidalis* (6x) (Chloridoideae, Zoysieae, *Sporobolus* sect. *Sporobolus*; v2) [49], *Zoysia japonica* (2n = 4x = 40; Chloridoideae, Zoysieae; version r1.1) [61], *Oropetium thomaeum* (2n = 2x = 20; Chloridoideae, Cynodonteae; v2.1) [45], *Eragrostis tef* (2n = 4x = 40; Chloridoideae, Eragrostideae; v3) [41], *Sorghum bicolor* (2n = 2x = 10; Panicoideae; v3.1) [62], and *Oryza sativa* (2n = 2x = 24; Oryzoideae; v7) [63]. *O. sativa*, *S. bicolor* and *O. thomaeum* did not undergo further WGD since the *rho* paleo-duplication and therefore represent excellent models for understanding the evolution of the ancestral Poaceae genome.

### Detection of homologous and putative orthologous regions

Orthologous protein sequences across the selected Poaceae genomes were identified using OrthoFinder2 (v2.5.2) [64] with default parameters. We also included *Setaria italica* [65] and *Brachypodium distachyon* [66], representing more divergent Poaceae lineages, to improve orthogroup inference by increasing phylogenetic signal and facilitating the identification of conserved orthologs. Because of high gene duplication rates in plants – particularly in polyploids – Reciprocal Best Blast Hit (RBBH) may under-detect homologous genes [67]. To mitigate this, we applied a more permissive Reciprocal Blast Hits (RBH) approach using BLASTn (BLAST, v2.9.0) [68] on CDS sequences with the following thresholds: e-value < 1e-5, identity ≥ 70%, alignment length ≥ 60 bp, and removal of CDSs with > 25% repetitive content. This RBH-based approach, implemented using the WGD_Tracker RBBH pipeline [69] (S1 Fig), maximized homolog detection between the sect. *Spartina* genomes (*S. maritimus*, *S. alterniflorus*) and seven Poaceae genomes.

### Divergence between homologous genes

Homologous gene pairs were aligned with MACSE (v2.05) [70,71], using default parameters. Synonymous substitution rates (Ks) were estimated using the Nei and Gojobori model [72] implemented in *codeml* (PAML v4.9) [73]. To reduce noise from misalignments or saturation, Ks values below 0.01 (highly similar copies) and>=3 (saturated divergence) were filtered out before subsequent analyses. Ks distributions were modeled using expectation-maximization fitting of Gaussian mixtures via the normalmixEM() function in the mixtools R package (v1.2.0) [74], with a convergence criterion set to 0.001 and 1,000 bootstrap iterations. All steps, from alignment to Ks modeling, are incorporated into WGD_Tracker’s Ks pipeline [69] (S1 Fig). Divergence times (T) were computed as: $\text{T}=\frac{\text{Ks}}{\text{2}\;\times \;\left(\text{6}.\text{5}\;\times {\text{ 10}}^{-\text{9}}\right)}$, assuming a monocot-specific synonymous substitution rate of 6.5 × 10 ⁻ ^9^ substitutions/site/year [75]. This substitution rate was selected as it represents the commonly used reference of Monocots substitution rate and is widely applied in grasses [76–80].

### Synteny search

To identify conserved syntenic blocks, we applied the WGD_Tracker’s synteny search pipeline [69] (S1 Fig), integrating filtered RBH pairs and their corresponding Ks values. Genes with multiple homologous copies on the same chromosome and Ks values outside the range 0.01 ≤ Ks < 3 were excluded. Syntenic blocks were defined as segments containing ≥ 5 orthologous gene pairs with no more than 100 intervening genes. This flexible criterion allowed detection of WGD-related blocks while minimizing false positives from local duplications. To assess the robustness of this parameter choice, synteny analyses were also performed using more stringent gap thresholds, and the results are presented in S2 Fig.

The JCVI tool [81] was used to generate a graphical representation of syntenic blocks between the *O. sativa*, *O. thomaeum* and *S. maritimus* karyotypes. Synteny between *S. alterniflorus* chromosomes and *S. maritimus* scaffolds allowed us to reconstruct *“pseudochromosomes”* in *S. maritimus*. The *S. maritimus “pseudochromosomes”* were numbered the same way as *S. alterniflorus* chromosomes.

### Karyotype reconstruction

Ancestral origin of *S. maritimus* genes was inferred from synteny across five genomes (*O. sativa*, *S. bicolor*, *E. tef*, *O. thomaeum*, and *Z. japonica*). A total of 12 ancestral chromosomes were defined using the *O. sativa* genome as the primary reference (Table 1). For each comparative hit, the chromosomal location in the compared species was translated into one or more putative ancestral chromosomes using the chromosome homology table (Table 1, columns 1–6). This procedure resulted, for each *Sporobolus* sect. *Spartina* gene, in a list of possible ancestral origins.

**Table 1:** Homology detected by RBH and dot-plot comparisons between chromosomes of *Oryza sativa*, *Sorghum bicolor*, *Eragrostis tef*, *Oropetium thomaeum*, *Zoysia japonica*, *Sporobolus alterniflorus* and scaffolds of *Sporobolus maritimus*. The columns labeled “Copy 1” to “Copy 4” are used to distinguish the four copies forming a chromosome composed from one or several scaffolds of *S. maritimus*. Ancestral grass chromosomes are represented in the first column [29,82]. Fig 4 illustrates the chromosomal homologies and rearrangements shown in this table, with a color code based on the 12 ancestral grass chromosomes.

Ancestral Grass Chromosomes	*Oryza sativa*	*Sorghum bicolor*	*Eragrostis tef*	*Oropetium thomaeum*	*Zoysia japonica*	*Sporobolus alterniflorus*	*Sporobolus maritimus*			
**Copy 1**	**Copy 2**	**Copy 3**	**Copy 4**
A1	1	3	3A, 3B	3	5, 6	2, 5, 3, 8	3	16, 21	9	15, 45, 50
A2	2	4, 1	1A, 1B	1	7, 8	9, 13, 6, 15	1	2	7	12, 26
A3	3	1	4A, 4B	4	1, 2	4, 7, 1, 12	4, 56	60, 43, 18	20, 22	64, 14, 19
A4	4	6	7A, 7B	6	11, 12	23, 26, 25, 30	51, 44, 37	47, 57, 59, 46	53, 29	52, 24
A5	5	9	9A, 9B	5	17, 18	1, 12, 18, 22	4	43, 40, 49	10	23, 39
A6	6	2, 10	2A, 2B	2	19, 20	14, 21, 10, 11	8	36, 25	5	41, 30, 62, 38, 54
A7	7	2	5A, 5B	7	3, 4	19, 27, 24, 28	48, 17, 63	27, 55, 42	32, 35	34, 28
A8	8	7	8A, 8B	9	13, 14	16, 17, 20, 29	6	11, 61	13	66, 33, 31
A9	9	2, 10	2A, 2B	2	19, 20	14, 21, 10, 11	8	36, 25	5	41, 30, 62, 38, 54
A10	10	4, 1	1A, 1B	1	7, 8	9, 13, 6, 15, 4	1	2	7	12, 26
A11	11	5	6A, 6B	8	9, 10	16, 17, 7, 31	6	11	20, 22	14, 19
A12	12	8	10A, 10B	10	15, 16	19, 27, 6, 4	48, 17	27, 55, 42	7	12, 26

Empirical probabilities for each ancestral origin were calculated by summing alignment scores (bitscores) supporting that origin and by normalizing with the total bitscore across all comparative hits for a given gene. Bootstrap replicates (n = 1000) were then generated by resampling ancestral origin labels with replacement according to these empirical probabilities.

The ancestral origin with the highest bootstrap support was considered the most likely one. An origin was retained as the unique assignment for a gene if its bootstrap support – the proportion of bootstrap replicates in which it was the majority – was at least 0.5 and exceeded the support of the next most probable origin by at least 0.05. If no origin clearly met these criteria, multiple origins with similar support were retained, and the gene was considered to have an ambiguous ancestral assignment.

Homologous gene blocks were cross-validated through synteny analysis between *S. maritimus* and *S. alterniflorus*, using a maximum intergene gap of 20 genes. Validated blocks were required to share a consistent ancestral identity across all comparisons. When a gene pair exhibited multiple equally supported ancestral origins, the final assignment was resolved by comparison with the dominant ancestral origin of neighboring syntenic pairs within the block. If the dominant block-level origin was among the potential origins of the ambiguous gene pair, this origin was assigned. In contrast, when a high proportion of genes within a genomic region displayed ambiguous ancestral origins, the syntenic block was terminated. Such regions were considered as transition zones, potentially reflecting chromosomal rearrangements that obscure reliable inference of a single ancestral origin.

The entire karyotype reconstruction process, as described above, is incorporated within WGD_Tracker Karyotype pipeline [69] (S1 Fig).

## Results

Comparative genomic analyses included seven species from major Poaceae lineages, diverging at varying evolutionary timescales from *Sporobolus* sections *Sporobolus* and *Spartina* (Fig 1; S1 Table). Orthologous conserved genomic blocks were inferred through a combination of homology searches, divergence estimations and synteny analysis. *Sporobolus* sect. *Spartina* karyotypes were then reconstructed following synteny detection, using ancestral chromosome assignments inferred for each gene.

### Detection of homologous regions

OrthoFinder2 was used to identify 42,707 orthogroups across the selected genomes. Of these, 15,671 contained at least one homologous protein sequence from each of the following: *Sporobolus maritimus*, *S. alterniflorus*, a close relative within Zoysieae (*Zoysia japonica*, *S. stapfianus*, or *S. pyramidalis*), a more divergent Chloridoideae (*Eragrostis tef* or *Oropetium thomaeum*), a Panicoideae (*Sorghum bicolor* or *Setaria italica*), and a BOP clade species (*Brachypodium distachyon* or *Oryza sativa*) (S3 Fig).

### Estimation of synonymous divergence (Ks) among homologous genes

**Fig 2:**
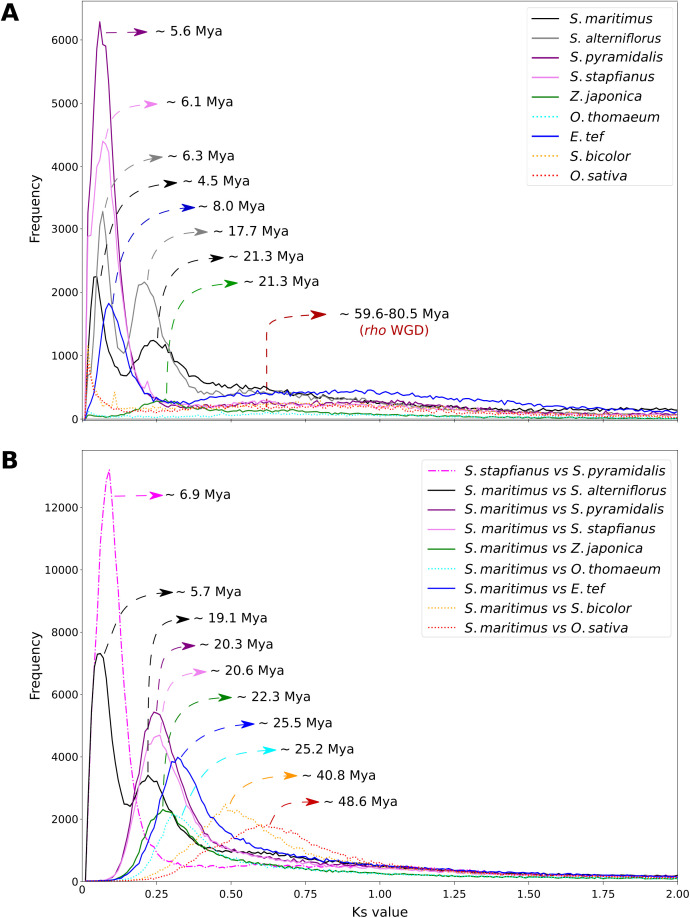
Distribution of synonymous substitution rates (Ks). (A) intragenomic comparisons within selected Poaceae genomes, and (B) intergenomic comparisons between *S. maritimus* and the other Poaceae genomes. Divergence times corresponding to each Ks peak were estimated using the mixtools R package and are displayed for both datasets.

Intraspecific Ks analyses revealed a shared peak across grass species with Ks values ranging from 0.775 to 1.046, corresponding to the well-established *rho* WGD event in Poaceae, dated between 38.0 and 107.5 Mya (Fig. 2A; Table 2). Species such as *E. tef*, *Z. japonica*, and members of the *Sporobolus* genus (*S. stapfianus*, *S. pyramidalis*, *S. maritimus*, and *S. alterniflorus*) exhibited additional Ks peaks that correspond to recent WGD events (Fig 2A; Table 2).

**Table 2:** Intra- and inter-specific Ks values and divergence time estimations. The Nei and Gojobori model [72] was used to estimate synonymous substitution rates (Ks). Divergence times were estimated using 6.5 x 10^−9^ substitutions per site per year [75]. The *rho* WGD refers to the Poaceae ancient polyploidization; WGD indicates more recent, lineage-specific duplication events; WGD1 and WGD2 correspond to the two successive WGD events identified in the section *Spartina*.

Comparisons		Ks		Divergence time (Mya)		
		mode	stdevs	min	mode	max
Intraspecific						
*O. sativa*	*rho* WGD	0.914	0.335	44.5	70.3	96.1
*S. bicolor*	*rho* WGD	0.971	0.343	48.3	74.7	101.1
*E. tef*	WGD	0.104	0.044	4.6	8.0	11.4
	*rho* WGD	1.006	0.345	50.8	77.4	103.9
*O. thomaeum*	*rho* WGD	1.011	0.309	54.0	77.8	101.5
*Z. japonica*	WGD	0.277	0.106	13.2	21.3	29.5
	*rho* WGD	0.884	0.272	47.1	68.0	88.9
*S. stapfianus*	WGD	0.080	0.042	2.9	6.1	9.4
	*rho* WGD	1.037	0.326	54.7	79.7	104.8
*S. pyramidalis*	WGD	0.072	0.039	2.6	5.6	8.6
	*rho* WGD	1.046	0.352	53.4	80.5	107.5
*S. maritimus*	WGD2	0.058	0.022	2.8	4.5	6.2
	WGD1	0.277	0.100	13.7	21.3	29.0
	*rho* WGD	0.879	0.279	46.2	67.6	89.1
*S. alterniflorus*	WGD2	0.082	0.024	4.4	6.3	8.1
	WGD1	0.230	0.066	12.6	17.7	22.7
	*rho* WGD	0.775	0.281	38.0	59.6	81.2
Interspecific						
*S. maritimus vs O. sativa*		0.632	0.154	36.8	48.6	60.5
*S. alterniflorus vs O. sativa*		0.613	0.144	36.1	47.2	58.2
*S. maritimus vs S. bicolor*		0.530	0.123	31.3	40.8	50.2
*S. alterniflorus vs S. bicolor*		0.498	0.107	30.1	38.3	46.6
*S. maritimus vs E. tef*		0.332	0.080	19.3	25.5	31.7
*S. alterniflorus vs E. tef*		0.309	0.069	18.5	23.8	29.1
*S. maritimus vs O. thomaeum*		0.327	0.073	19.6	25.2	30.8
*S. alterniflorus vs O. thomaeum*		0.306	0.063	18.7	23.5	28.4
*S. maritimus vs Z. japonica*		0.291	0.073	16.7	22.3	28.0
*S. alterniflorus vs Z. japonica*		0.266	0.067	15.3	20.5	25.6
*S. maritimus vs S. stapfianus*		0.267	0.071	15.1	20.6	26.0
*S. alterniflorus vs S. stapfianus*		0.236	0.065	13.2	18.2	23.2
*S. maritimus vs S. pyramidalis*		0.263	0.070	14.9	20.3	25.6
*S. alterniflorus vs S. pyramidalis*		0.234	0.062	13.2	18.0	22.8
*S. maritimus vs S. alterniflorus*		0.074	0.026	3.7	5.7	7.7
		0.248	0.086	12.4	19.1	25.7
*S. stapfianus vs S. pyramidalis*		0.090	0.044	3.5	6.9	10.3

Interspecific Ks analyses between *Sporobolus* sect. *Spartina* genomes and all Poaceae representatives – from the rice to the *Sporobolus* sect. *Sporobolus* genomes – revealed single, distinct Ks peak, reflecting interlineage divergence events (Fig. 2B; S4 Fig; Table 2). The comparison between *S. stapfianus* and *S. pyramidalis* produced a Ks peak of ≈ 0.09, corresponding to a divergence window of 3.5–10.3 Mya, suggesting recent speciation within the *Sporobolus* lineage (Fig 2B; Table 2).

Intraspecific Ks distributions revealed bimodal peaks in *S. maritimus* (2.8–6.2 and 13.7–29.0 Mya) and *S. alterniflorus* (4.4–8.1 and 12.6–22.7 Mya), which likely correspond to two independent rounds of WGD within the section *Spartina*. Comparative Ks analysis between *S. maritimus* and *S. alterniflorus* estimated their divergence time between 3.7 and 7.7 Mya.

### Synteny search

**Fig 3:**
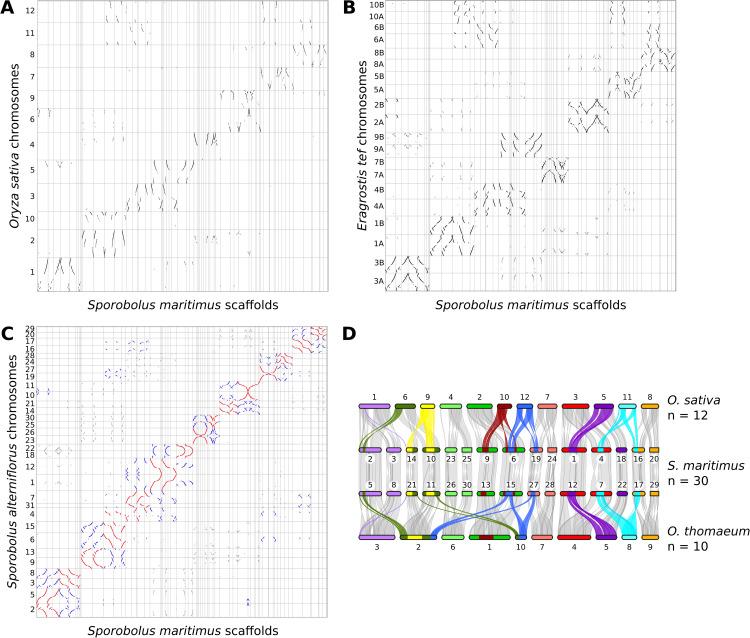
Graphical representation of homologous genes and conserved syntenic blocks. **(A)** Dotplot comparison between *Sporobolus maritimus* and *Oryza sativa* (N = 19,232); black dots indicate Ks values between 0.478–0.786, all others in grey. **(B)** Dotplot comparison between *S. maritimus* and *Eragrostis tef* (N = 58,411); black dots: Ks = 0.252–0.412, others in grey. **(C)** Dotplot comparison between *Sporobolus maritimus* and *Sporobolus alterniflorus* (N = 101,194); red (Ks = 0.048–0.100), purple (Ks = 0.100–0.162), blue (Ks = 0.162–334), others in grey. **(D)** Linear syntenic map of homologous blocks resulting from WGD2 within *S. maritimus “pseudochromosomes”*, compared with orthologous regions from *O. sativa* and *Oropetium thomaeum*. Note: *S. maritimus* scaffold ordering in dotplots follows the order provided in S2 Fig legend for reproducibility.

Comparative synteny analyses revealed a 1:4 chromosomal correspondence between diploid genomes (e.g., *O. sativa*, *S. bicolor*, and *O. thomaeum*) and those of *S. maritimus* and *S. alterniflorus*, consistent with two successive lineage-specific WGD events in sect. *Spartina* (Fig 3A; S5 Fig A and B; Table 1). Comparisons between tetraploid genomes (e.g., *E. tef* and *Z. japonica*) and the sect. *Spartina* showed a 2:4 ratio, whereas the comparison between *S. maritimus* and *S. alterniflorus* displayed a 4:4 ratio.

Several *S. maritimus* scaffolds exhibit homology and synteny with more than one chromosome of the Poaceae species compared (Table 1). For instance, scaffold 3 is fully syntenic with the rice chromosome 1, while it is only partially syntenic with the rice chromosome 6 (Fig 3A and S. *maritimus* pseudochromosome 2 in Fig 3D). Furthermore, rice chromosomes 2 and 10 are completely syntenic with *S. maritimus* scaffold 1 (Fig 3A and S. *maritimus* pseudochromosome 9 in Fig 3D). Similar observations were made in the *S. alterniflorus* genome (S5 Fig). These observations result from chromosomal rearrangements, including one translocation and seven fusion events that occurred during the evolutionary history of the *S. maritimus* and *S. alterniflorus* genomes (Fig 3; S5 Fig). Two of these events are shared with all the Chloridoideae genomes used in our comparisons, while the others are specific to *S. maritimus* and *S. alterniflorus* genomes.

### Karyotype evolution

**Fig 4:**
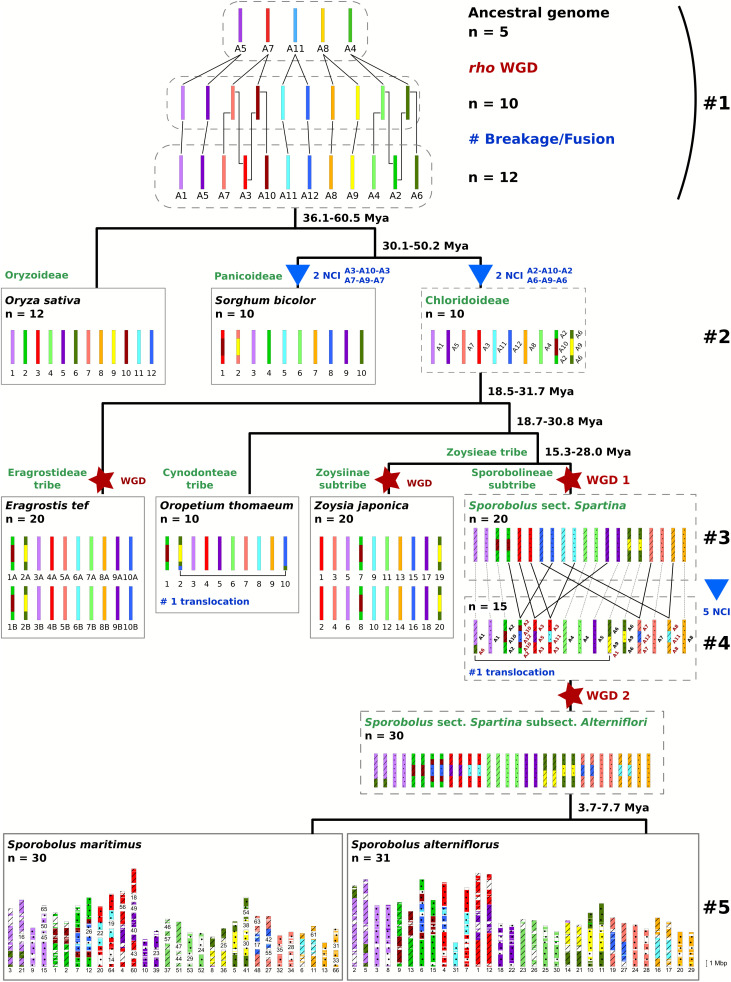
Evolution of the ancestral grass genome with a focus on the Chloridoideae subfamily and the *Sporobolus* section *Spartina.* A 12-color code was used to trace the chromosomal segments inherited from the ancestral Poaceae genome (n = 12) [29]. WGD = Whole Genome Duplication; NCI = Nested Chromosome Insertion. Colored dots and dashed lines denote homeologous chromosomes derived from WGD1 in *Sporobolus* sect. *Spartina*.

Building on the ancestral karyotype model of n = 5 [29,82], followed by a WGD and chromosomal rearrangements that produced the n = 12 Poaceae ancestor, we reconstructed the karyotypic evolution of *S. maritimus* and *S. alterniflorus* (Fig 4, sections 1–5). The search for synteny between the two *Sporobolus* sect. *Spartina* genomes resulted in 96,990 pairs of homologous and syntenic genes corresponding to 2,631 syntenic blocks with only one ancestral origin per block. From this information and dotplot comparisons, we deduced the genome history of both *S. maritimus* and *S. alterniflorus* by locating the different chromosomal rearrangement events in the evolutionary history of the *Sporobolus* sect. *Spartina* genomes. We observed two independent fusion events specific to Chloridoideae, leading to a genome with x = 10 (Fig 4, section 2). One event involved the integration of chromosome A10 between the arms of A2 (A2-A10-A2), whereas the other consisted of the insertion of A9 into A6 (A6-A9-A6). Then, the *Sporobolus* sect. *Spartina* ancestor underwent a first WGD, resulting in n = 20 (Fig 4, section 3). Subsequently, five fusion events: A2-A10-A12-A10-A2; A7-A12-A7; A3-A5-A3; A3-A11-A3; A8-A11-A8; and one translocation between the distal regions of A6-9 and A1, led to a genome with n = 15 (Fig 4, section 4). The second WGD event occurred in this genome, resulting in the modern *S. maritimus* genome with n = 30. The chromosomal rearrangement at the origin of the extra chromosomes pair in *S. alterniflorus* involves genomic regions homologous to the Poaceae ancestral chromosomes 11 and 12 (Fig 4, section 5).

## Discussion

This study reconstructs the evolutionary history of polyploid *Sporobolus* sect. *Spartina* genomes by tracing chromosomal rearrangements shaped by successive rounds of WGD and diploidization. Through comparative genomic analyses across representative Poaceae species, we distinguished shared from lineage-specific WGD events, and identified mechanisms of base chromosome number reduction.

We developed customizable pipelines dedicated to genomic comparisons allowing first, the identification of duplicated genes through homology and synteny searches, and second, to estimate the divergence between these duplicated genes and finally to reconstruct the karyotypes. Among investigated species, if the *rho* WGD appears as the only shared WGD event, we report here a shared WGD in *S. stapfianus* and *S. pyramidalis* genomes*,* and identified five NCIs between the two ancestral WGDs which formed *S. maritimus* and *S. alterniflorus* genomes, leading to their new basic number by descending dysploidy.

### WGD events among Chloridoideae

Shared and independent WGD events among the different species were analyzed, and divergence times between subgenomes were estimated. In polyploids, divergence times estimated between duplicated subgenomes may reflect the divergence between the parental species (in allo-polyploids) and/or the transition from polysomic (expected in auto-polyploids) to disomic inheritance [83]. Therefore, the estimated divergence time represents the upper age limit of the genome duplication events. Our analyzes are consistent with WGDs reported in previous studies, including the *rho* paleo-duplication shared by all Poaceae species [24,77], as well as the more recent allo-polyploidization events of *Eragrostis* tef [41] and *Zoysia japonica* [84] genomes.

In the *Sporobolus* sect. *Sporobolus*, we evaluated subgenome divergence time as ≈ 2.9–9.4 Mya in *S. stapfianus* and ≈ 2.6–8.6 Mya in *S. pyramidalis*. Our comparison between these two genomes revealed a single Ks peak, indicating a shared WGD event and an estimated divergence time between the two species to be around 3.5–10.3 Mya. However, *S. stapfianus* and *S. pyramidalis* are polyploid complexes considered as tetraploid and hexaploid species, respectively [49]. The reasons for the lack of an additional Ks peak in the hexaploid *S. pyramidalis* are still unclear. One possible hypothesis is the scenario of successive WGDs that may result in overlapping Ks peaks suggesting that both *Sporobolus* species formed within a short evolutionary time. Further genomic comparisons at the chromosome level would improve the detection of WGD, and assess divergence between duplicated chromosomes in *S. pyramidalis*.

Distributions of Ks values between homeologous gene pairs of the *Sporobolus* sect. *Spartina* genomes exhibited two additional peaks since the *rho* WGD. These polyploidization events are estimated to have occurred around 13.7–29.0 (WGD1) and 2.8–6.2 (WGD2) Mya, in *S. maritimus*, and 12.6–22.7 (WGD1) and 4.4–8.1 (WGD2) Mya, in *S. alterniflorus.* These estimates are similar to those previously reported for both *S. maritimus* and *S. alterniflorus* (9.6–24.4 and 2.1–6.2) [48].

Divergence times between *Sporobolus* sect. *Spartina* genomes and the selected grasses revealed a Ks peak of ≈ 0.61–0.63 and 0.50–0.53 between the section *Spartina* and the rice and the sorghum genomes, respectively. These values are consistent with those reported in previous studies comparing Chloridoideae genomes to (i) the rice genome: 0.58 [40], 0.64 [84]; and (ii) the sorghum genome: 0.42 [40]. The single Ks peaks in interspecific comparisons imply that observed WGDs are species-specific, and provide the first clues for independent WGDs in the section *Spartina*.

Comparisons between the section *Spartina* and the other Chloridoideae tribes estimated divergence times with the genomes of *E. tef* and *O. thomaeum* as 18.5–31.7 and 18.7–30.8 Mya, respectively. Divergence time estimates align with prior studies, such as the 23–44 Mya separation between Eragrostideae and Zoysieae [85] and the ~ 26–35 Mya divergence between Zoysieae and Cynodonteae [51,86]. However, Gallaher and colleagues [60] estimated slightly higher divergence times with ≈ 42.75 (Zoysieae *vs* Eragrostideae) and ≈ 36.82 Mya (Zoysieae *vs* Cynodonteae).

The Zoysieae Benth. tribe diversified into two subtribes: Sporobolineae (e.g., *Sporobolus sp.*) and Zoysiinae (e.g., *Zoysia japonica*). We estimated the divergence time between these two subtribes to have occurred ≈ 15.3–28.0 Mya which is consistent with the previous estimation of 14–25.23 Mya [60]. Within the Sporobolineae subtribe, we found that divergence between *Sporobolus* and *Spartina* sections occurred ≈ 13.2–26.0 Mya, in agreement with the estimates of ≈ 7–22 Mya [85]. Our analysis showed that no recent WGD events are shared between these two *Sporobolus* sections. The sect. *Sporobolus* is phylogenetically more distant from sect. *Spartina* than all the other sections of the *Sporobolus* genus: sect. *Fimbriatae*, sect. *Crypsis*, sect. *Triachyrum*, sect. *Virginicae*, sect. *Pyramidati*, sect. *Airoids*, sect. *Cryptandri*, sect. *Clandestini*, sect. *Calamovilfa* [50]. The WGD1 event identified in sect. *Spartina*, estimated at 12.6–29.0 Mya, postdated the divergence between the *Sporobolus* and *Spartina* sections and may potentially be shared with more closely related *Sporobolus* species.

In the sect. *Spartina*, chromosome numbers range from 2n = 4x = 40 to 2n = 124 [56], suggesting that it likely evolved from a tetraploid ancestor. Variable chromosome number is observed in the sect. *Clandestini*: *Sporobolus neglectus* and *S. clandestinus* with 2n = 36 [87–89]; *S. vaginiflorus* with 2n = 54 [90]; *S. compositus* with 2n = 54–108 [87,91]. This may reflect historical chromosomal rearrangements and shifts in basic number from 10 to 6 or 9, possibly mirroring those observed in sect. *Spartina*. The sect. *Calamovilfa,* which diverged 12–20 Mya from sect. *Spartina*, as indicated from chloroplast genome data [51]*,* is divided into two subsections. In the subsect. *Calamovilfa*, chromosome counts of 2n = 40, 60 have been observed in *Calamovilfa longifolia* [92,93] and *C. gigantea* [93,94], while in the subsect. *Floridani*, 2n = 72 and 2n = 30 chromosomes were reported in *Sporobolus heterolepis* [88,95] and *S. interruptus* [96], respectively. This suggests that the ancestor of the sect. *Calamovilfa* kept a basic chromosome number of 10, which evolved differently in the two subsections. These patterns warrant targeted comparative genomics to confirm whether WGD1 is shared across sister sections such as *Clandestini* and *Calamovilfa*.

### Ancestral grass genome evolution in *Sporobolus*

The comparative analysis of the *S. maritimus* (2n = 60) and *S. alterniflorus* (2n = 62) genomes revealed the chromosome rearrangements responsible for their chromosome number difference [48]. The present study provides evidence that the segment of chromosome 15, now inserted into the center of *S. alterniflorus* chromosome 4, shares homology with the ancestral grass chromosome 12 (in dark blue, Fig 4). Additionally, *S. alterniflorus* chromosome 31, which originated from a segment of chromosome 4, is homologous to the ancestral chromosome 11 (in light blue, Fig 4). This chromosomal rearrangement occurred after all reported WGD events and led to speciation between *S. maritimus* and *S. alterniflorus* that diverged 3.7–7.7 Mya.

Although *S. maritimus* and *S. alterniflorus* were previously regarded as hexaploids (2n = 6x = 60; considering a basic chromosome number x = 10 [56]), synteny with diploid and tetraploid grasses reveals a 1:4 and 2:4 homology ratio, respectively, suggesting an octoploid origin followed by diploidization. Chromosome restructuring reduced the basic number from n = 20 to n = 15 prior to a subsequent WGD (WGD2), supporting their classification as diploidized meso-octoploids [48].

We found that several *S. maritimus* scaffolds and *S. alterniflorus* chromosomes showed homology and synteny with multiple chromosomes of the same Poaceae species, revealing a total of seven nested chromosome insertions (NCI) and one translocation in the evolutionary history of this genome prior WGD2.

Two NCI events (A2-A10-A2 and A6-A9-A6) are discernable when comparing the sect. *Spartina* genomes with genomes outside the Chloridoideae subfamily (*O. sativa* and *S. bicolor*). These fusion events likely occurred in the Chloridoideae ancestor, as these rearrangements are observed in all four copies of the sect. *Spartina* genomes and are shared among the various Chloridoideae genomes examined in this study. Our findings are consistent with previous studies that found a chromosome number reduction from 12 to 10 in the ancestor of Chloridoideae, following these same two chromosomal fusion events [42,44,46].

The remaining five NCIs (A3-A5-A3; A3-A11-A3; A8-A11-A8; A7-A12-A7; A2-A10-A12-A10-A2) observed in only two of four sect. *Spartina* genome copies likely occurred after WGD1 but before WGD2, reshaping the genome and reducing the base chromosome number from 20 to 15 (descending dysploidy). This new odd basic chromosome number (x = 15) would have been fertile in a 2n = 30 plant but has never been recorded in natural populations. The strong similarity and karyotype structure between duplicated copies from WGD2 (copies 1 and 2) would suggest auto-polyploidy or allo-polyploidy between slightly divergent parental genomes for this polyploidization event, which led to an ancestral genome with 2n = 4x = 60, and confirms that the reported chromosomal restructurations were rapidly followed by WGD2 [48]. The modern tetraploid *S. maritimus* genome is still displaying 2n = 4x = 60 chromosomes, while the *S. alterniflorus* genome was affected by a new restructuration leading to 2n = 4x = 62 chromosomes.

This study sheds light on the importance of NCIs in the diploidization process affecting polyploid genome evolution. NCIs result from fusion between two non-homologous chromosomes by the insertion of one chromosome between the arms of the second one [97]. This descending dysploidy mechanism requires at least three double strand-breaks and that the inserted chromosome centromere remains functional [98]. Several studies have highlighted that NCIs are considered as important mechanisms in the grass family, with reported events in Pooideae (*Aegilops tauschii* [35], *Brachypodium distachyon* [99], *Secale cereale* [100]), Panicoideae (*Setaria italica* [101], *Zea mays* [102], *Eremochloa ophiuroides* [103], *Saccharum* complex [104]) and Chloridoideae (*Zoysia japonica* [46], *Cynodon dactylon* [42], *Cynodon transvaalensis* [43], *Eleusine coracana* [44]). Although the mechanisms enabling NCI are still poorly understood, these types of chromosomal rearrangements are common in grasses, strongly suggesting that grass chromosomes possess some intrinsic genomic characteristics that predispose them to NCI [97].

In this study, we identified that the ancestral chromosome 12 (A12) is involved in two NCIs (A2-A10-A12-A10-A2 and A7-A12-A7), as well as in the chromosomal rearrangement that led to the extra-chromosome pair in *S. alterniflorus*. Other studies have highlighted NCIs involving the A12 as the inserted chromosome in *Eleusine coracana* (A5-A12-A5) [44] and in *Cynodon transvaalensis* (A1-A12-A1) [43]. Chromosome 12 appears also involved in chromosomal rearrangement in *Cynodon dactylon* [42] and in a translocation with A6 in *Oropetium thomaeum* [45]. The frequent involvement of ancestral chromosomes 12 in NCIs across Chloridoideae lineages, including multiple events identified in this study, suggests a possible structural or sequence predisposition. Investigating the genomic architecture of A12 could unveil novel mechanisms governing chromosome fusion dynamics in grasses.

This study reconstructs the complex history of polyploidization and chromosomal rearrangement in the *Sporobolus* sect. *Spartina*. We confirm two major WGD events (WGD1 and WGD2), separated by a suite of chromosomal rearrangements, including one translocation and five lineage-specific NCIs, which reduced the base chromosome number from n = 20 to n = 15. These structural innovations culminated in the extant karyotypes of *S. maritimus* (2n = 60) and *S. alterniflorus* (2n = 62). Our findings reinforce the importance of NCIs as drivers of descending dysploidy and genome plasticity in grasses. They offer new perspectives for understanding the chromatinian context of genomic regions subject to NCIs. Our results also pave the way for future studies regarding the functional consequences of genome restructuring and fractionation and their adaptive consequences in these ecologically important but still understudied grass species.

## Supporting information

S1 Fig Workflow used for genome analyses and comparisons. This consists of four main steps: (1) detection of homologous sequences using a Reciprocal Blast Hit approach or the OrthoFinder2 tool; (2) estimations of gene pair divergence times by calculating synonymous substitution rates; (3) identification of syntenic blocks; and (4) karyotype reconstruction.
(TIFF)

S2 Fig Effect of parameter stringency on the detection of syntenic blocks. Dotplot comparisons between *Sporobolus maritimus* and *Oryza sativa* using different synteny search settings. Syntenic blocks are defined as following: ≥ 5 orthologous gene pairs, and intervening genes between syntenic pairs **(A)** ≤ 100 (as presented in Fig 3A), **(B)** ≤ 20. **(C)** Table summarizing the detected number of CDS in both species and the number of syntenic gene pairs under three settings. *S. maritimus* scaffolds are ordered as following in all dotplots: 3, 21, 16, 9, 15, 45, 50, 58, 65, 1, 2, 7, 12, 26, 20, 22, 64, 14, 19, 4, 56, 60, 43, 40, 49, 18, 10, 39, 23, 37, 57, 59, 46, 51, 44, 47, 53, 29, 52, 24, 8, 36, 25, 5, 41, 30, 62, 38, 54, 48, 17, 63, 27, 55, 42, 32, 35, 34, 28, 6, 11, 61, 13, 66, 33, 31, 67, 68, 69.
(TIFF)

S3 Fig Venn diagram representing the number of orthogroups shared among analyzed genomes. (TIFF)

S4 Fig Distribution of the estimated synonymous substitution (Ks) rates between *S. alterniflorus* and the selected Poaceae genomes. The divergence time of each peak (based on the mode estimated using the R package *mixtools*) is presented on the Ks distribution.
(TIFF)

S5 Fig Graphical representations of homologous genes and conserved syntenic blocks. Dotplot comparisons between *Sporobolus maritimus* and **(A)** *Sorghum bicolor* (N = 21,779), **(B)** *Oropetium thomaeum* (N = 29,876), **(C)** *Zoysia japonica* (N = 36,660), **(D)** *Sporobolus stapfianus* (N = 6,654), **(E)** *Sporobolus pyramidalis* (N = 26,737). Dots are colored in black when Ks values are within the following ranges: 0.407–0.653 **(A)**, 0.253–0.400 **(B)**, 0.218–0.364 **(C)**, 0.196–0.338 **(D)**, 0.193–0.333 **(E)**. All others dots are in grey.
(TIFF)

S1 Table Chromosome number, ploidy level, genome size and repeat content estimations for the species analyzed for Reciprocal Blast Hits and synteny searches. (DOCX)
