# Supplementary figures and images for: Recurrent polyploidy and descending dysploidy as plant genome shapers: Insights from Sporobolus (Chloridoideae, Poaceae) genomes
Source: PLoS One. 2026 Feb 23;21(2):e0343073. doi: 10.1371/journal.pone.0343073 (PMC12928440; doi:10.1371/journal.pone.0343073)

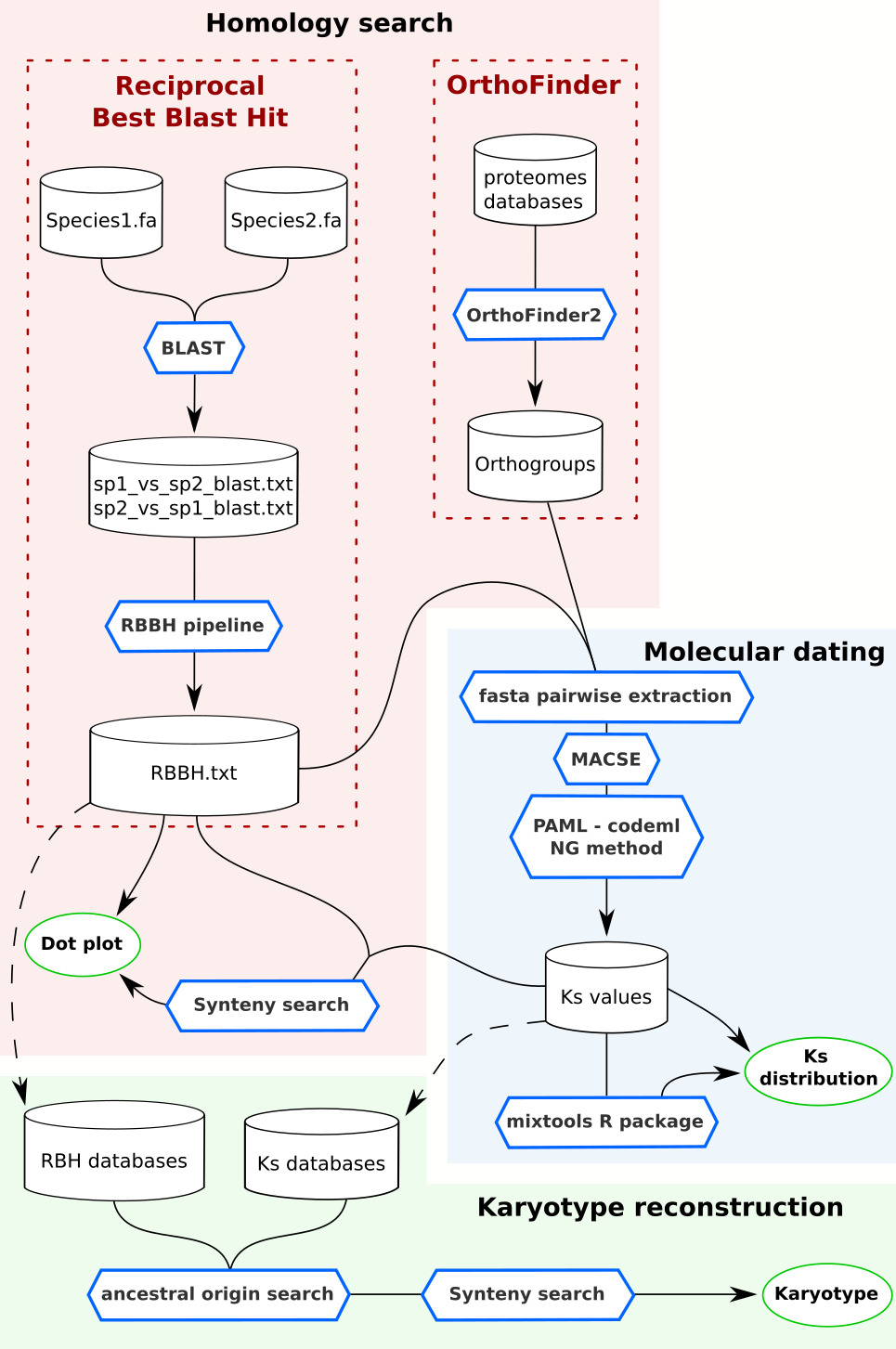

Supplement: S1 Fig — This consists of four main steps: (1) detection of homologous sequences using a Reciprocal Blast Hit approach or the OrthoFinder2 tool; (2) estimations of gene pair divergence times by calculating synonymous substitution rates; (3) identification of syntenic blocks; and (4) karyotype reconstruction. (TIFF) [file pone.0343073.s001.tiff]

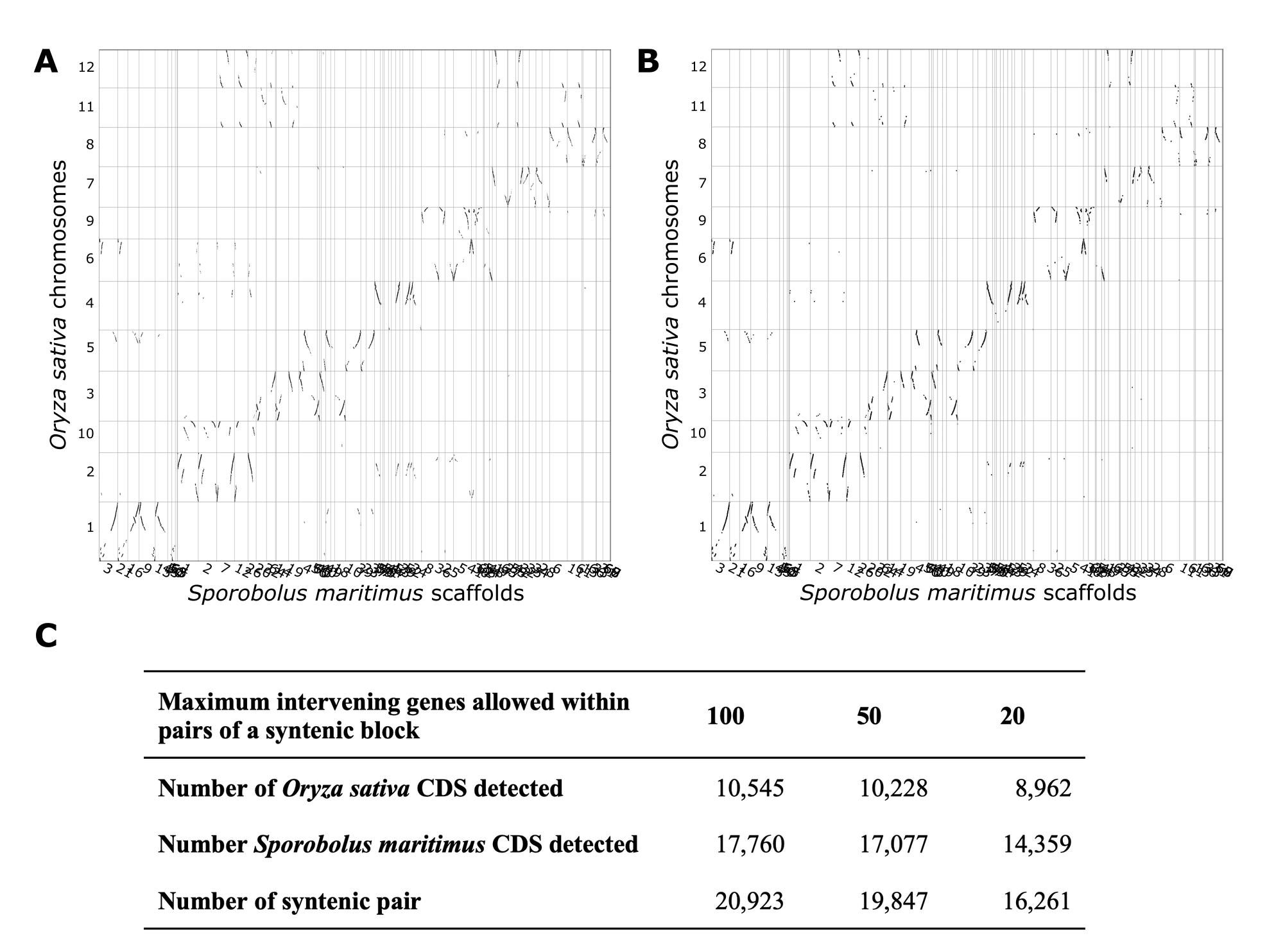

Supplement: S2 Fig — Dotplot comparisons between Sporobolus maritimus and Oryza sativa using different synteny search settings. Syntenic blocks are defined as following: ≥ 5 orthologous gene pairs, and intervening genes between syntenic pairs (A) ≤ 100 (as presented in Fig 3A), (B) ≤ 20. (C) Table summarizing the detected number of CDS in both species and the number of syntenic gene pairs under three settings. S. maritimus scaffolds are ordered as following in all dotplots: 3, 21, 16, 9, 15, 45, 50, 58, 65, 1, 2, 7, 12, 26, 20, 22, 64, 14, 19, 4, 56, 60, 43, 40, 49, 18, 10, 39, 23, 37, 57, 59, 46, 51, 44, 47, 53, 29, 52, 24, 8, 36, 25, 5, 41, 30, 62, 38, 54, 48, 17, 63, 27, 55, 42, 32, 35, 34, 28, 6, 11, 61, 13, 66, 33, 31, 67, 68, 69. (TIFF) [file pone.0343073.s002.tiff]

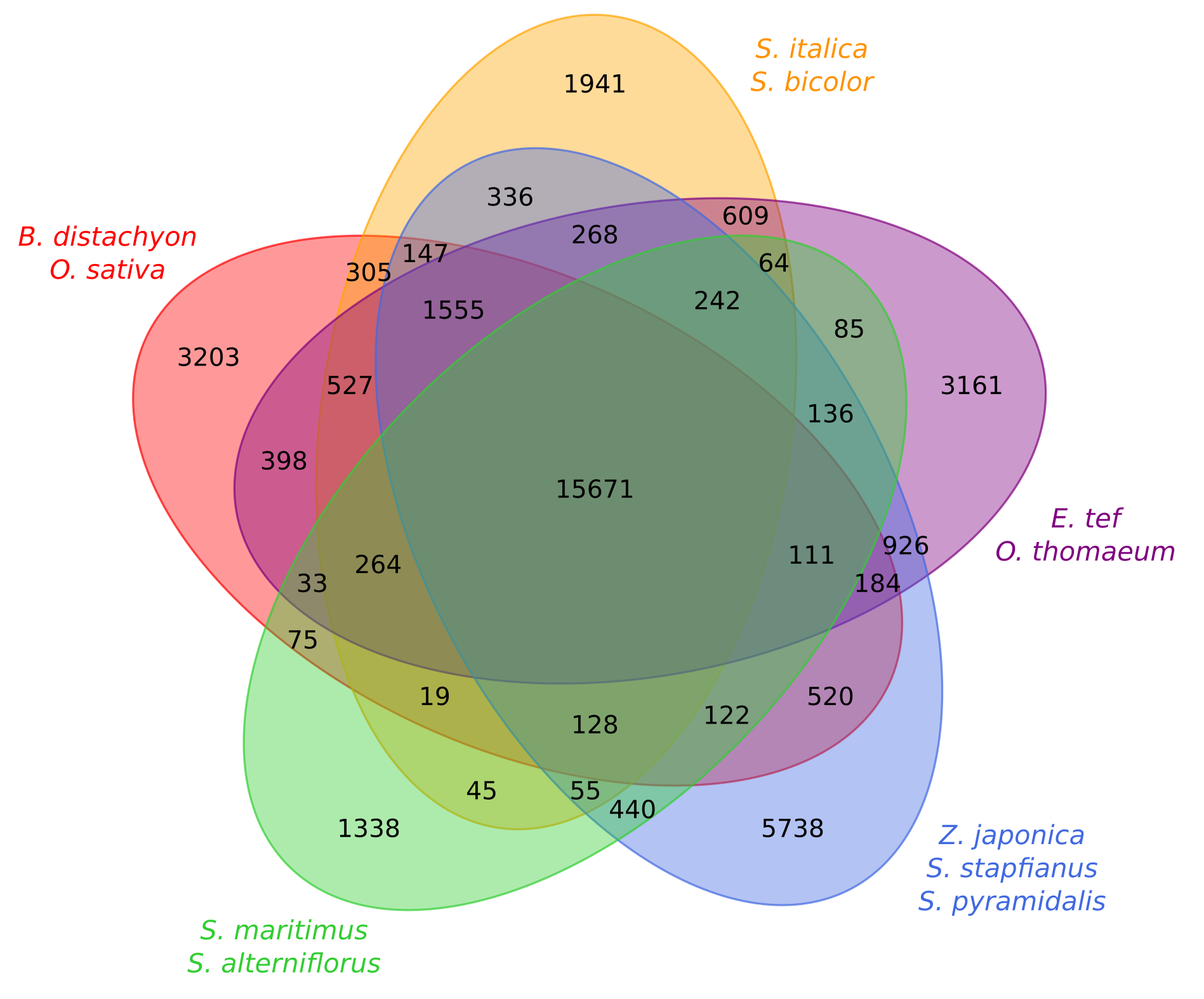

Supplement: S3 Fig — (TIFF) [file pone.0343073.s003.tiff]

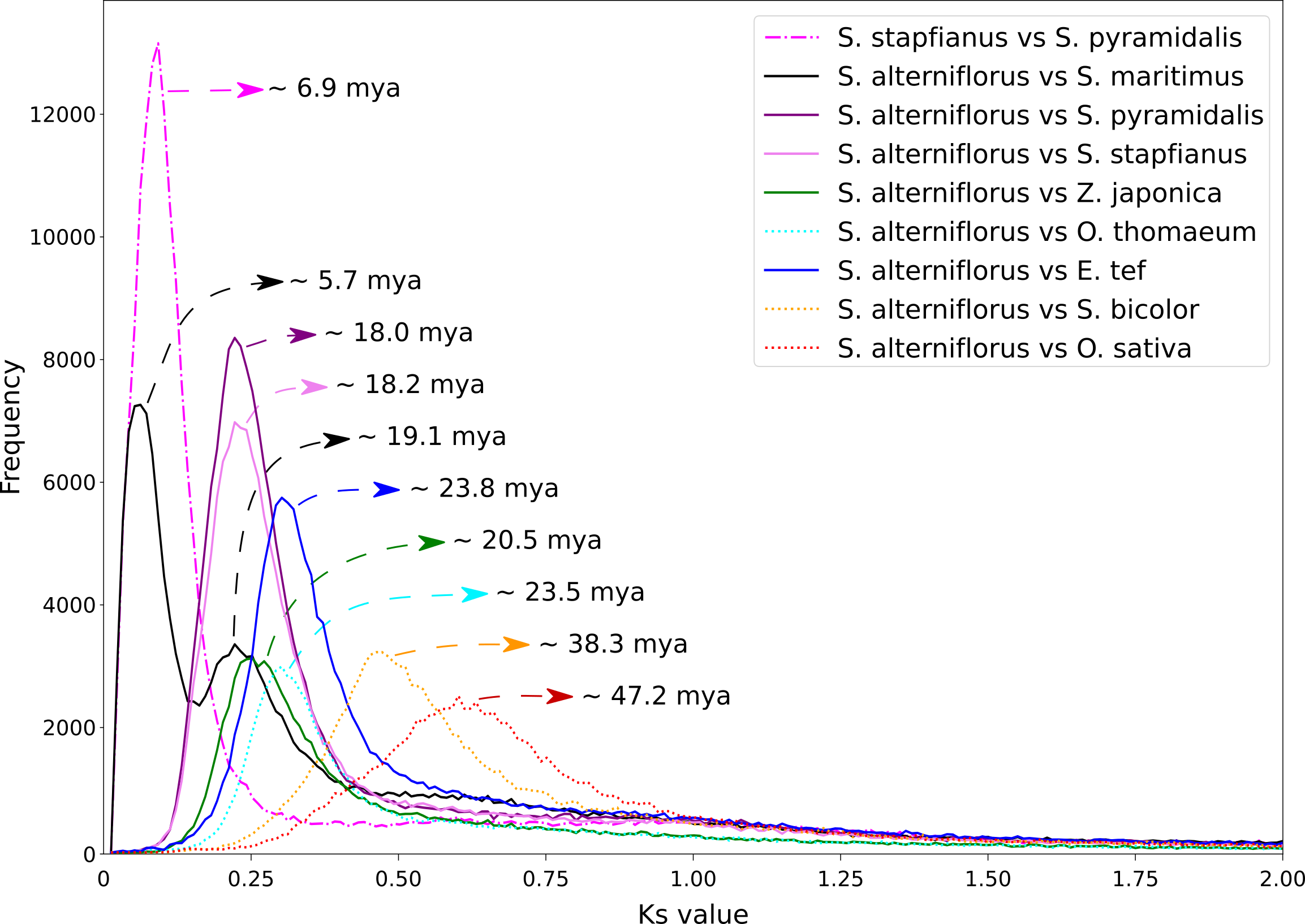

Supplement: S4 Fig — The divergence time of each peak (based on the mode estimated using the R package mixtools) is presented on the Ks distribution. (TIFF) [file pone.0343073.s004.tiff]

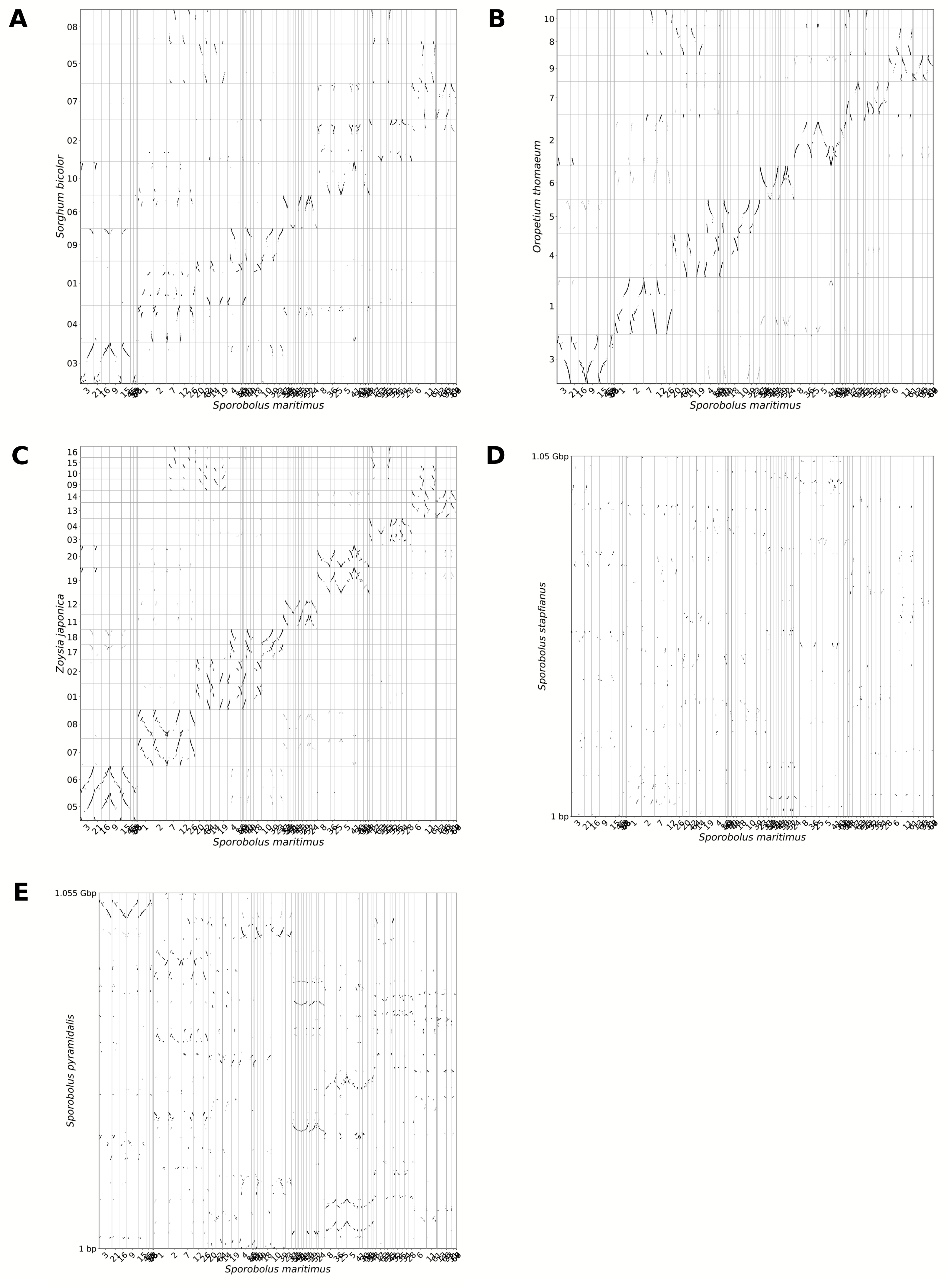

Supplement: S5 Fig — Dotplot comparisons between Sporobolus maritimus and (A) Sorghum bicolor (N = 21,779), (B) Oropetium thomaeum (N = 29,876), (C) Zoysia japonica (N = 36,660), (D) Sporobolus stapfianus (N = 6,654), (E) Sporobolus pyramidalis (N = 26,737). Dots are colored in black when Ks values are within the following ranges: 0.407–0.653 (A), 0.253–0.400 (B), 0.218–0.364 (C), 0.196–0.338 (D), 0.193–0.333 (E). All others dots are in grey. (TIFF) [file pone.0343073.s005.tiff]
