# Supplementary material for: Recurrent polyploidy and descending dysploidy as plant genome shapers: Insights from Sporobolus (Chloridoideae, Poaceae) genomes
Source: PLoS One. 2026 Feb 23;21(2):e0343073. doi: 10.1371/journal.pone.0343073 (PMC12928440; doi:10.1371/journal.pone.0343073)
Supplement: S1 Table — (DOCX) [file pone.0343073.s006.docx]

**S1 Table.** **Chromosome number, ploidy level, genome size and repeat content estimations for the species analyzed for Reciprocal Blast Hits and synteny searches.**

| **Species** | **Ploidy level** | **Chromosome number** | **Genome size (Mb)** | **Repeated elements (%)** | **Assembled genome** | | |
| --- | --- | --- | --- | --- | --- | --- | --- |
|  |  |  |  |  | **size (Mb)** | **protein-coding genes number** | **version** |
| *Sporobolus maritimus* | 4x | 60 | 1,795 ^[12]^ | 59.3 ^[15]^ | 1,595 | 79,568 | v1 ^[15]^ |
| *Sporobolus alterniflorus* | 4x | 62 | 1,956 ^[12]^ | 73.1 ^[15]^ | 1,630 | 73,711 | v1 ^[14]^ |
| *Sporobolus pyramidalis* | ~ 6x | 24, 30, 36 ^[1, 3, 4]^ | 1,867 ^[13]^ | 41.3 ^[13]^ | 1,055 | 51,207 | v2 ^[13]^ |
| *Sporobolus stapfianus* | ~ 4x | 24, 36 ^[2, 3]^ | 1,354 ^[13]^ | 39.7 ^[13]^ | 1,080 | 52,808 | v2 ^[13]^ |
| *Zoysia japonica* | 4x | 40 | 390 ^[10]^ | 40.9 ^[10]^ | 334 | 49,103 | v r1.1 ^[10]^ |
| *Oropetium thomaeum* | 2x | 20 | 250 ^[7]^ | 43.0 ^[7]^ | 236 | 28,835 | v2.1 ^[8]^ |
| *Eragrostis tef* | 4x | 40 | 622 ^[9]^ | 25.6 ^[9]^ | 576 | 68,255 | v3 ^[9]^ |
| *Sorghum bicolor* | 2x | 20 | 730 ^[5]^ | 62.0 ^[5]^ | 709 | 34,129 | v3.1.1 ^[11]^ |
| *Oryza sativa* | 2x | 24 | 389 ^[5]^ | 39.5 ^[5]^ | 380 | 42,189 | v7.0 ^[6]^ |

^[1]^ Moffett AA, Hurcombe R. Chromosome numbers of South African grasses. Heredity. 1949;3(3):369–73.

^[2]^ de Wet JMJD, Anderson LJ. Chromosome Numbers in Transvaal Grasses. Cytologia (Tokyo). 1956;21(1):1–10.

^[3]^ de Wet JMJ. Chromosome Numbers and Some Morphological Attributes of Various South African Grasses. Am J Bot. 1960;47(1):44–9.

^[4]^ Tateoka T. Chromosome Numbers of Some East African Grasses. Am J Bot. 1965;52(8):864–9.

^[5]^ Oliver KR, McComb JA, Greene WK. Transposable Elements: Powerful Contributors to Angiosperm Evolution and Diversity. Genome Biol Evol. 2013;5(10):1886–901.

^[6]^ Kawahara Y, De La Bastide M, Hamilton JP, Kanamori H, McCombie WR, Ouyang S, et al. Improvement of the *Oryza sativa* Nipponbare reference genome using next generation sequence and optical map data. Rice. 2013;6(1):4.

^[7]^ VanBuren R, Bryant D, Edger PP, Tang H, Burgess D, Challabathula D, et al. Single-molecule sequencing of the desiccation-tolerant grass *Oropetium thomaeum*. Nature. 2015;527(7579):508–11.

^[8]^ VanBuren R, Wai CM, Keilwagen J, Pardo J. A chromosome‐scale assembly of the model desiccation tolerant grass *Oropetium thomaeum*. Plant Direct. 2018;2(11):e00096.

^[9]^ VanBuren R, Wai CM, Wang X, Pardo J, Yocca AE, Wang H, et al. Exceptional subgenome stability and functional divergence in the allotetraploid Ethiopian cereal teff. Nat Commun. 2020;11(1):884.

^[10]^ Tanaka H, Hirakawa H, Kosugi S, Nakayama S, Ono A, Watanabe A, et al. Sequencing and comparative analyses of the genomes of zoysiagrasses. DNA Res. 2016;23(2):171–80.

^[11]^ McCormick RF, Truong SK, Sreedasyam A, Jenkins J, Shu S, Sims D, et al. The *Sorghum bicolor* reference genome: improved assembly, gene annotations, a transcriptome atlas, and signatures of genome organization. Plant J. 2018;93(2):338–54.

^[12]^ Giraud D, Lima O, Huteau V, Coriton O, Boutte J, Kovarik A, et al. Evolutionary dynamics of transposable elements and satellite DNAs in polyploid *Spartina* species. Plant Sci. 2021;302:110671.

^[13]^ Chávez Montes RA, Haber A, Pardo J, Powell RF, Divisetty UK, Silva AT, et al. A comparative genomics examination of desiccation tolerance and sensitivity in two sister grass species. Proc Natl Acad Sci. 2022;119(5):e2118886119.

^[14]^ Hao Y, Wang XF, Guo Y, Li TY, Yang J, Ainouche ML, et al. Genomic and phenotypic signatures provide insights into the wide adaptation of a global plant invader. Plant Commun. 2024;100820.

^[15]^ Salmon A, Hao Y, Milin M, Lima O, Cavé-Radet A, Giraud D, et al. On the way to diploidization and unexpected ploidy in the grass *Sporobolus* section *Spartina* mesopolyploids. Nat Commun. 2025;16(1):1997.
